# Evaluating haptic experience using EEG and deep learning across multiple modalities: linking stimulus and self-reports

**DOI:** 10.3389/fnins.2026.1666558

**Published:** 2026-01-30

**Authors:** Haneen Alsuradi, Yonas Atinafu, Mohamad Eid

**Affiliations:** 1 Engineering Division, New York University Abu Dhabi, Abu Dhabi, United Arab Emirates; 2 Science Division, New York University Abu Dhabi, Abu Dhabi, United Arab Emirates

**Keywords:** cognitive-interface, deep-learning, EEG, haptics, self-report

## Abstract

Conventionally, evaluations of haptic interfaces have relied on self-reported assessments, which offer limited objectivity and can disrupt the user experience, making it challenging to design interfaces that dynamically adapt to users' cognitive state in real time. To overcome these limitations, cognitive haptic interfaces leverage neurophysiological measures such as EEG and deep learning to directly capture the brain's responses to haptic stimulation. A key challenge is how to label these neural responses: do we ground models in objectively controlled Physical Stimulation (PS) parameters, or in participants' Self-Reported (SR) perceptions? The goal of this work is not to demonstrate that EEG can reproduce subjective reports, but rather to systematically examine how neural responses relate to these two aspects of haptic experience by training deep learning models under both PS and SR labeling schemes. Here, we investigate how PS- versus SR-based labeling impacts model performance across four modalities: (i) delayed force-feedback (DFF), (ii) fingertip vibration feedback (FVF), (iii) upper-body vibration feedback (UVF), and (iv) fingertip thermal feedback (FTF). We evaluate three deep learning benchmarked architectures: ATCNet, EEG Inception, and EEG Conformer on EEG data labeled according to both approaches. Across all modalities, PS-labeled models yield more stable and higher performance than SR-labeled models in a group-level leave-one-subject-out (LOSO) setting, with the largest gains at near-perceptual-threshold levels (e.g., mild thermal changes, moderate vibration intensities, borderline delay settings) where SR labels are most variable across individuals. Rather than aiming to replace self-reports, these results reveal when EEG-based models align more closely with the physical stimulation than with participants' reports and support using PS-trained decoders as a structured first-stage representation that can later be adapted with user-specific SR information.

## 1 Introduction

The human sense of touch is fundamental to everyday experience, providing information about object properties, environmental hazards, and social cues (Fulkerson, 2013; Gallace and Spence, 2010). Over the past decade, there has been a growing trend to integrate various forms of haptics, such as kinesthetic, vibrotactile, and thermal (Huang et al., 2022; Eid and Al Osman, 2015), into a wide spectrum of applications like rehabilitation (Liou et al., 2024), virtual reality (Fisch et al., 2003), gaming (Yun et al., 2023), and teleoperation (González et al., 2021). This integration aims to enhance the realism and immersiveness of the user experience (Gani et al., 2022).

The evaluation of the user experience is crucial for the advancement of haptic technologies. Real-time evaluation during haptic interactions enables interface adaptation, allowing for better personalization to individual preferences (Peck and Childers, 2003). The classical approach to characterizing the human haptic experience is through self-reporting and/or behavioral observations (Alsuradi et al., 2020a). Self-reporting evaluates users' haptic experience after the termination of a haptic interaction. This method is subjective, memory-dependent (Berka et al., 2007; Kivikangas et al., 2011), and could be influenced by social pressure (Picard, 2000). Behavioral observation, while offering real-time feedback, indirectly infers the haptic experience by measuring observable actions. This implicative nature limits its accuracy, as it cannot directly capture the user's internal cognitive and emotional states, leading to potential misinterpretation of the actual experience. Consequently, it remains difficult to design haptic interfaces that adapt online to a user's internal state without repeatedly interrupting the interaction for self-reports.

Neurohaptics, defined as the evaluation of haptic experience through cognitive analysis of neurophysiological signals, has been on the rise as an alternative to traditional self-report methods, particularly through cognitive haptic interfaces (Alsuradi et al., 2020a). This method is based on capturing the neural traces exhibited in response to a haptic stimulation and automatically decoding it using trained machine learning models. Electroencephalography (EEG) offers a direct view into rapid neural dynamics through its high temporal resolution and cost-effectiveness compared to other brain imaging methods, such as fMRI or MEG (Alsuradi et al., 2020a). The EEG-based approach has been applied to evaluate haptic experience in various contexts, including detecting tactile feedback on touchscreen devices (Alsuradi et al., 2020b), classifying textures (Eldeeb et al., 2019, 2020), assessing thermal perception (Shan and Yang, 2020), and recognizing grasping tasks (Bodda and Diwakar, 2022). These approaches primarily rely on deep learning methods that aim to extract neural traces relevant to the haptic experience for classification.

However, despite the promise of EEG for decoding neural responses to haptic stimulation, a fundamental methodological question persists: how should these EEG data be labeled for machine learning training? Broadly, two labeling schemes exist. One approach is based on Physical Stimulation (PS), in which labels are derived from the experimenter-controlled parameters of the haptic feedback, such as vibration intensity, or temperature values. This approach ensures consistency and objectivity while minimizing the risk of human error during labeling. Nonetheless, PS labels do not account for individual differences in perception; participants may experience the same temperature value (e.g., 40 °C) differently based on factors like skin sensitivity, prior exposure, or personal tolerance levels. The second labeling scheme uses Self-Reported (SR) labels, in which participants directly communicate their thoughts and feelings through interviews, surveys or questionnaires (Braga Rodrigues et al., 2020). SR labels can capture important personal nuances and subject-specific thresholds, but they risk introducing human error. Borderline stimuli, such as a mild temperature change or a moderately intense vibration (e.g., weak vs. very weak), may be labeled inconsistently if participants are distracted or uncertain.Thus, PS and SR labels highlight different aspects of haptic experience: PS labels primarily reflect the stimulus-driven component, whereas SR labels capture the perception-driven component.

In this work, our goal is not to show that EEG can perfectly reproduce subjective reports, but to systematically compare how neural responses relate to these two aspects of haptic experience by training deep learning models under PS vs. SR labeling schemes. We use this comparison to identify when self-reports closely follow the physical stimulation and when they diverge from it. Such divergence, often around near-threshold conditions or more subjective experiences, points to strong individual differences and uncertainty, making group-level SR-labeled models less reliable in those circumstances. In these cases, PS-trained models act as a stable first stage that can later be adapted with SR data from a single user, whereas when SR and PS largely agree, PS-trained models alone may be sufficient for a cognitive haptic interface. We conduct an extensive analysis of the implications of the two labeling schemes, PS and SR, providing a more comprehensive evaluation across four distinct haptic experiences:

- **Delayed force-feedback (DFF):** investigating haptic *delay* in force-feedback (Haneen Alsuradi and Eid, 2022),
- **Fingertip vibration feedback (FVF):** exploring the effect of different vibration *intensities* at the fingertip (Wanjoo Park and Eid, 2021),
- **Upper-body vibration feedback (UVF):** exploring varying *urgency* levels conveyed through different upper-body vibration patterns (Wanjoo Park and Eid, 2024),
- **Fingertip thermal feedback (FTF):** studying discrete *temperature* classes ranging from very cold to very hot within the non-harmful range (9 °C to 42 °C) (Wanjoo Park and Eid, 2023).

By comparing four haptic modalities, we also identify which haptic experiences are more reliably decoded from EEG and which are only weakly expressed in the neural signals. Each dataset is characterized by distinct stimulus mechanisms, with varying levels of: delay for force-feedback, vibration intensity for fingertip experience, urgency using upper-body vibrations, and temperature for thermal stimulation. We employed three bench-marked deep learning models, packaged in the braindecode toolbox (Schirrmeister et al., 2017), ATCNet, EEG Inception, and EEG Conformer, to classify EEG data from these modalities. We compare the performance of these models under PS versus SR labels using leave-one-subject-out (LOSO) cross-validation method.

The remainder of this paper is organized as follows. Section II provides a detailed description of our experimental setups, including how each of the four haptic datasets was gathered, how we analyzed PS versus SR labeling consistency, the details of the deep learning architectures, EEG preprocessing, and training schemes. Section III presents classification results, including both overall metrics and per-class analyses. Section IV offers a discussion of our core findings and the broader implications of labeling schemes for EEG-based cognitive haptic interfaces. Finally, Section V concludes with final remarks and outlines potential directions for future research in this emerging area.

## 2 Methods

This section details the apparatus and protocols used to acquire EEG data across four haptic experiences: (1) Delayed force-feedback, (2) Fingertip vibration feedback, (3) Upper-body vibration feedback, and (4) Fingertip thermal feedback. Although each experience employed specific haptic interfaces, participants wore multi-channel EEG caps (32 or 64 electrodes), and performed tasks designed to elicit neural responses to the corresponding haptic stimuli.

Furthermore, despite differences in the experimental setups and protocols, the underlying EEG processing pipeline was uniform to ensure consistency. All EEG data were recorded at a sampling rate of 1 kHz and underwent the same steps for preprocessing. First, EEG data were band-pass filtered between 0.1 to 40Hz removing slow drifts and high-frequency artifacts, commonly practiced in literature (Kingphai and Moshfeghi, 2021; Kang et al., 2021). Then, notch-filtered at 50/60 Hz for power line noise suppression. This was followed by common average referencing while at the same time retaining the EEG data of FCz channel which is the online reference channel. EEG data were then downsampled to 250 Hz; this is to reduce the computational cost while maintaining sufficient temporal resolution. We opted for this minimal processing to mimic the condition of EEG data generally obtained in online cognitive haptic interfaces, where a classification outcome is required in real-time (Delorme, 2023). Preprocessed time series were epoched around the corresponding key haptic events as described in the following subsections. All preprocessing was conducted with code written in MATLAB version 2022a (MathWorks, United States) and the EEGLAB toolbox (v2021.0).

For each dataset, the PS labels were obtained from the actual delivered physical stimulation while the SR labels were elicited from participants after each trial. We defined SR labels independently from PS labels to preserve the distinction between objective stimulation parameters and subjective perceptual experience, allowing participants to report what they truly perceived without being constrained by predefined categories. We later align PS and SR labels for the purpose of quantifying correspondence or divergence between the two domains, providing insight into how physical stimuli are perceived rather than enforcing equivalence between the label spaces.

Each dataset employed a unique set of participants, ensuring no overlap between the groups. Below, we describe the core apparatus, number of participants, and task protocols for each dataset.

### 2.1 Delayed force-feedback

Researchers have increasingly studied how delays in force-feedback systems affect user performance in mediated manipulation scenarios (Haneen Alsuradi and Eid, 2022; Alsuradi et al., 2021, 2022). Understanding haptic delay is critical to ensure responsiveness and realism in human–machine interaction. The perception of haptic delay has been examined in psychophysical studies (Fu et al., 2018). For example, work on delayed force feedback shows that temporal delays distort perceived mechanical properties such as stiffness, mass, and damping, directly impacting the user's perceptual haptic interactions. Even small delays can disrupt sensorimotor integration, leading to degraded task performance and reduced user trust in the system. The goal of this dataset was to examine the EEG correlates of delay perception and to assess how the brain detects and encodes delays during force-feedback interaction.

#### 2.1.1 Apparatus and participants

A force-feedback device (Geomagic Touch haptic device) was employed to deliver force-feedback to participants during a pick-and-drop task. Participants could feel the weight of the object picked, and a haptic delay was introduced as the object is released in a bucket. A total of 34 healthy adults participated (right-handed, normal or corrected-to-normal vision). Exclusion criteria include participants below the age of 18 and/or left-handed with reported traumatic brain injuries, neural abnormalities, and/or muscle atrophy. EEG recordings (64-channel cap) followed the international 10–20 layout, with ground at FPz and online reference at FCz. The study protocol was reviewed and approved by the Institutional Review Board of New York University Abu Dhabi (Approval No. HRPP-2021-17).

#### 2.1.2 Experimental protocol

**Figure 1:**
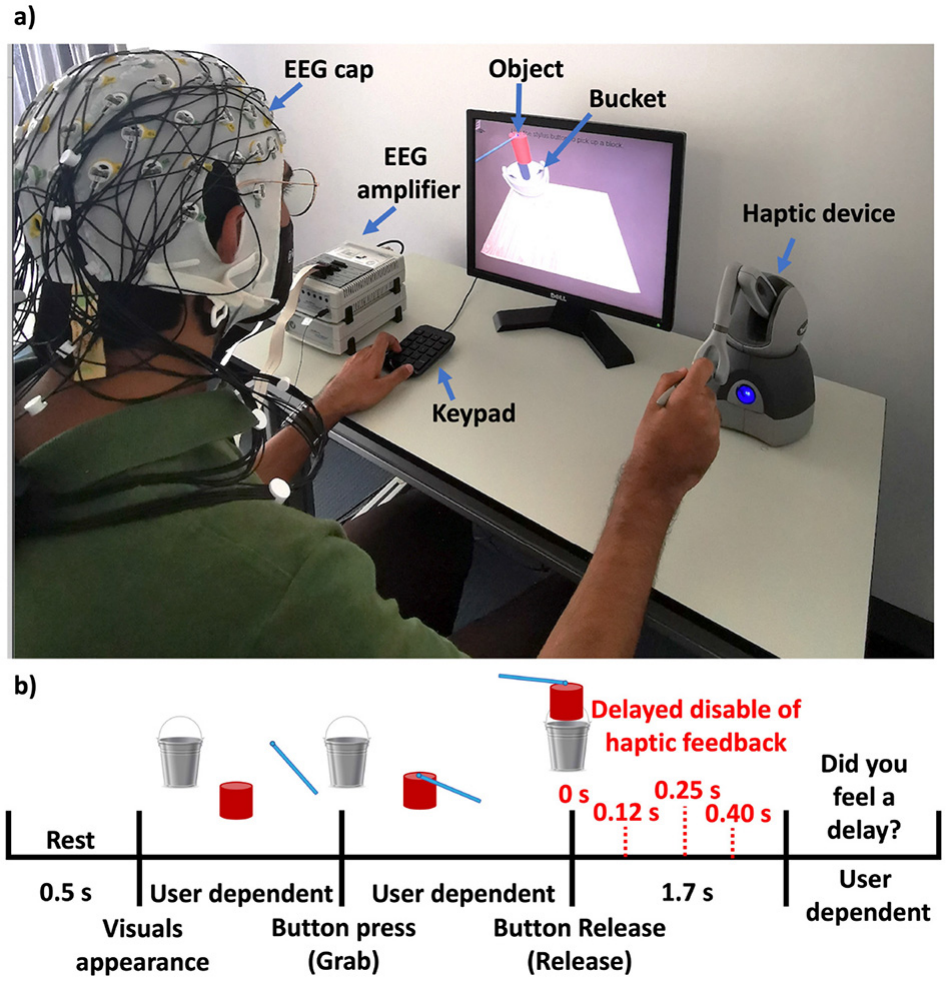
**(a)** Experimental setup of the DFF experience. **(b)** Task and protocol of the DFF experience.

As illustrated in Figure 1a, the participants were seated on a desk, facing a monitor that showed a virtual environment. They gripped the stylus with their right hand. The primary task was *pick-and-place*; a single trial of the task is shown in Figure 1b and consisted of the following:

- Pick-up: Participants used the stylus to grasp a virtual object on-screen and move it to a target location (bucket).
- Perception of weight: During object manipulation, the weight of the object is delivered through the haptic device immediately after the object is picked-up.
- Release: Upon object release, the weight of the object persisted for an additional period of time which involved four levels of haptic delay: 0 ms, 120 ms, 250 ms, or 400 ms. These values were selected based on a psychophysical study, which showed that they span the relevant psychophysical range for perceiving haptic delay; the perception threshold is around 85 ms (Haneen Alsuradi and Eid, 2022).
- Self-report: At the end of the trial, participants indicated whether they perceived any delay (*no delay* vs. *delay*).
- The process was repeated for a predetermined number of tests (40 trials for each of the four conditions), ensuring balanced coverage of the four delay levels.

EEG data were epoched around the actual release of the object. For additional details on task design and neural analysis, we refer the readers to (Haneen Alsuradi and Eid 2022).

#### 2.1.3 Dataset

We aligned the number of conditions for the PS and SR labels to ensure a fair comparison when evaluating the performance of deep learning models under both labeling systems. PS labels consist of four categories (0 ms, 120 ms, 250 ms, and 400 ms), while the SR labels have two categories: *no delay* and *delay*. To align the number of categories, all PS labels greater than 0 ms (120 ms, 250 ms, and 400 ms) were converted to the *delay* category, while 0 ms was mapped to the *no delay* category, resulting in a binary classification.

### 2.2 Fingertip vibration feedback

Vibration intensity plays a key role in conveying tactile information, especially in wearable and assistive technologies. Accurate perception and modulation of vibration can impact motor control, attention, and the usability of haptic interfaces in real-world tasks. The perception of vibration intensity has been studied in psychophysical experiments; For example, a study using fingertip vibration showed that perceived intensity varies not only with amplitude and frequency but also with stimulus duration (Bochereau et al., 2014). Furthermore, neural processes during the experience of various vibration intensities at the fingertip were previously explored (Wanjoo Park and Eid, 2021). The objective of this dataset is to evaluate the vibrotactile intensity (no vibration, low vibration intensity, and high vibration intensity) based on EEG neural signatures.

#### 2.2.1 Apparatus and participants

A vibrotactile actuator was attached to the participant's fingertip (left index). Three distinct intensity levels were set via micro-controller: no vibration, low vibration intensity (~1.5 g), high vibration intensity (~2.3 g). A total of 29 participants were recruited for this study, wearing a 32-channel EEG system with FCz as a reference electrode. Exclusion criteria were a person with orthopedic hand conditions or with a history of neurological or psychiatric disease. The study protocol was reviewed and approved by the Institutional Review Board of New York University Abu Dhabi (Approval No. HRPP–2020–80).

#### 2.2.2 Experimental protocol

**Figure 2:**
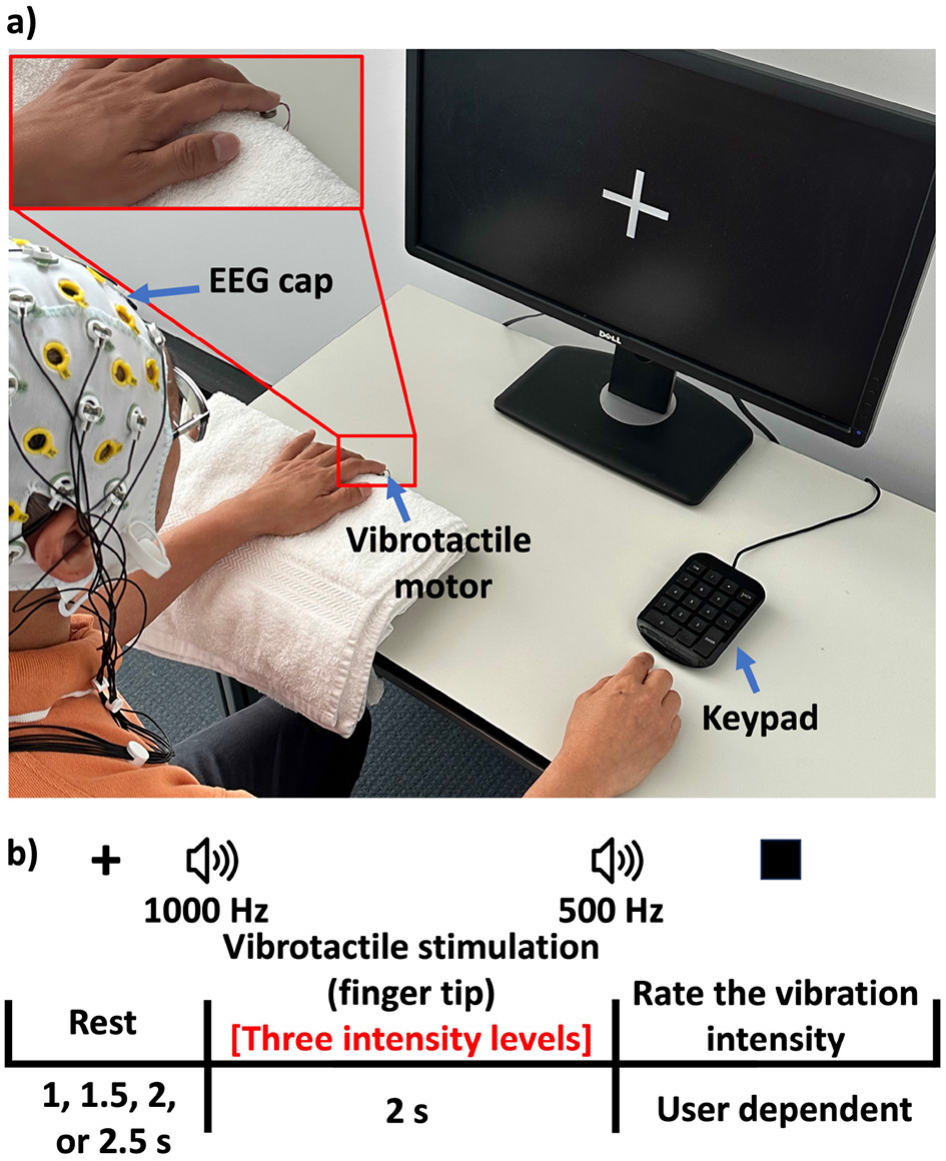
**(a)** Experimental setup of the FVF experience. **(b)** Task and protocol of the FVF experience.

Figure 2a shows the experimental setup for this experiment. The timeline of a single trial of the task is shown in Figure 2b:

- Rest: Participants rested their left hand on a stable surface (table), minimizing movement artifacts.
- Vibration: At the onset of each trial, one of the three vibration levels was randomly selected and delivered to the participant's fingertip. The three vibration levels [no vibration, low-intensity vibration (1.56 g), and high-intensity vibration (2.26 g)], were selected based on psychophysical testing confirming that these specific intensities were clearly distinguishable by participants (Wanjoo Park and Eid, 2021).
- Self-report: Participants rated the perceived vibration intensity (no vibration, very weak, weak, strong, and very strong).
- The process was repeated for a predetermined number of trials (100 trials for each of the three conditions), ensuring balanced coverage of the three vibration intensity levels.

EEG data were epoched around the onset of the vibration at the fingertip. For additional details on the task design and neural analysis, we refer the readers to (Wanjoo Park and Eid 2021).

#### 2.2.3 Dataset

PS labels consist of three categories (*no vibration, low vibration intensity*, and *high vibration intensity*), while the SR labels have five categories (*no vibration, very weak, weak, strong*, and *very strong*). To align the categories, SR-labeled epochs were mapped as follows: *no vibration* remains as is, *very weak* and *weak* to *low vibration intensity*, and *strong* and *very strong* to *high vibration intensity*. This resulted in a three-class classification for this experience.

### 2.3 Upper-body vibration feedback

Communicating urgency through haptics is critical in scenarios where visual or auditory channels are overloaded or unavailable. Effective haptic encoding of urgency can improve situational awareness, and safety in high-stakes environments such as driving, aviation, or assistive systems. In this dataset, the goal was to explore whether vibration patterns on the torso of varying intensity and spatial distribution encode distinct urgency levels (Wanjoo Park and Eid, 2024).

#### 2.3.1 Apparatus and participants

A wearable haptic vest (bHaptics TactSuitX40) equipped with 40 vibration motors distributed over the chest and abdomen areas was used for delivering vibration feedback to the upper-body. A total of 31 participants participated in this study, wearing a 64-channel EEG system with FCz as a reference electrode. Exclusion criteria were being under 18 or having a history of traumatic brain injury, neurological disorders, or muscle atrophy. The study protocol was reviewed and approved by the Institutional Review Board of New York University Abu Dhabi (Approval No. HRPP-2022-96).

#### 2.3.2 Experimental protocol

**Figure 3:**
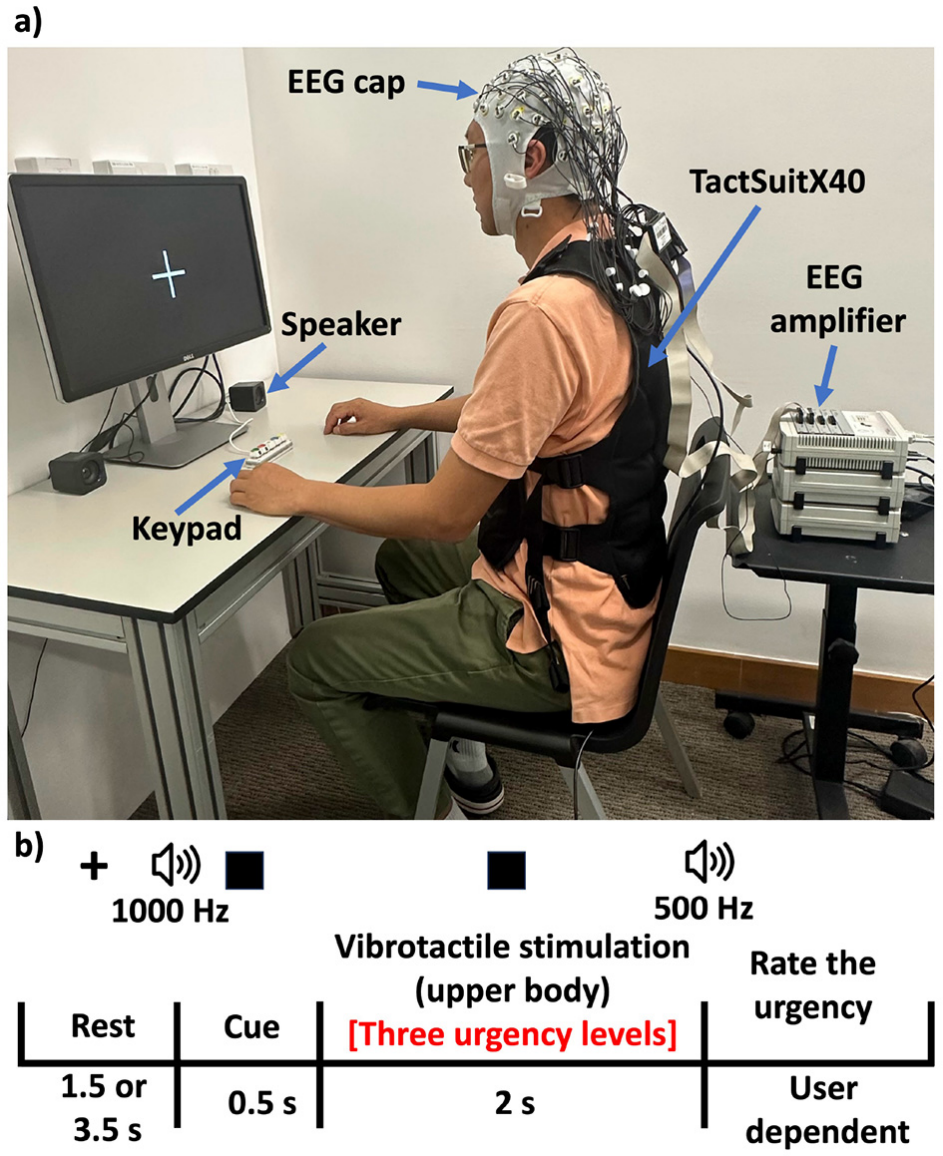
**(a)** Experimental setup of the UVF experience. **(b)** Task and protocol of the UVF experience.

The experimental setup is shown in Figure 3a and a single trial of the task followed the timeline shown in Figure 3b. We describe the task below:

- Rest: 1.5–3.5 s of rest.
- Vibrotactile stimulation: 2 s of one of three stimulation conditions: no vibration pattern (no urgency), urgent vibration pattern (low urgency), very urgent vibration pattern (high urgency). The urgent vibration pattern used 4 actuators activated for 550 ms with an intensity of 0.39 g, whereas the very urgent pattern used 20 actuators activated for 300 ms with an intensity of 1.96 g. These specific patterns were selected based on psychophysical testing reported in our previous work that ensured they are significantly distinguishable (Elsaid et al., 2022).
- Self-report: Participants used a keypad to rate urgency (no urgency, very low urgency, low urgency, high urgency, and very high urgency).
- The process was repeated for a predetermined number of trials (36 trial for each of the three conditions), ensuring balanced coverage of the three urgency patterns.

EEG data were epoched around the onset of the vibrotactile stimulation. For additional details on the task design and neural analysis, we refer the readers to (Wanjoo Park and Eid 2024).

#### 2.3.3 Dataset

PS labels consist of three categories (*no urgency, low urgency*, and *high urgency*), while the SR labels have five categories (*no urgency, very low urgency, low urgency, high urgency*, and *very high urgency*). To align the categories, SR-labeled epochs were mapped as follows: *no urgency* remains as is, *very low urgency* and *low urgency* to *low urgency*, and *high urgency* and *very high urgency* to *high urgency*. This resulted in a three-class classification for this experience.

### 2.4 Fingertip thermal feedback

Temperature-based cues are important for conveying non-urgent information such as comfort, status changes, or environmental conditions. Thermal haptics is particularly valuable in wearable devices, assistive technologies, and ambient feedback systems where less intrusive, persistent signaling is required. Thermal actuation offers a slower and more subtle form of haptic stimulation compared to force-feedback or vibration. The perception of thermal sensation has been studied in psychophysical experiments (Lee, 2025). For example, foundational work in cutaneous thermal perception shows that perceived warmth and cold depend not only on absolute temperature but also on the rate of temperature change, skin type, initial skin temperature, and geographical background of the person. EEG correlates of discrete temperature levels applied to the fingertip were previously explored (Wanjoo Park and Eid, 2023).

#### 2.4.1 Apparatus and participants

A custom-built thermal display utilized thermoelectric modules (Peltiers) to provide four distinct temperature levels: 9 °C, 15 °C, 32 °C, and 42 °C (Wanjoo Park and Eid, 2023). The device's real-time feedback loop maintained ±1.5°C accuracy. A total of 28 participants took part in this study and wore a 64-channel EEG cap, with ground at FPz and reference at FCz. Exclusion criteria include participants below the age of 18, being left-handed, having a history of traumatic brain injury, neurological disorders, or muscle atrophy. The study protocol was reviewed and approved by the Institutional Review Board of New York University Abu Dhabi (Approval No. HRPP–2020–80).

#### 2.4.2 Experimental protocol

**Figure 4:**
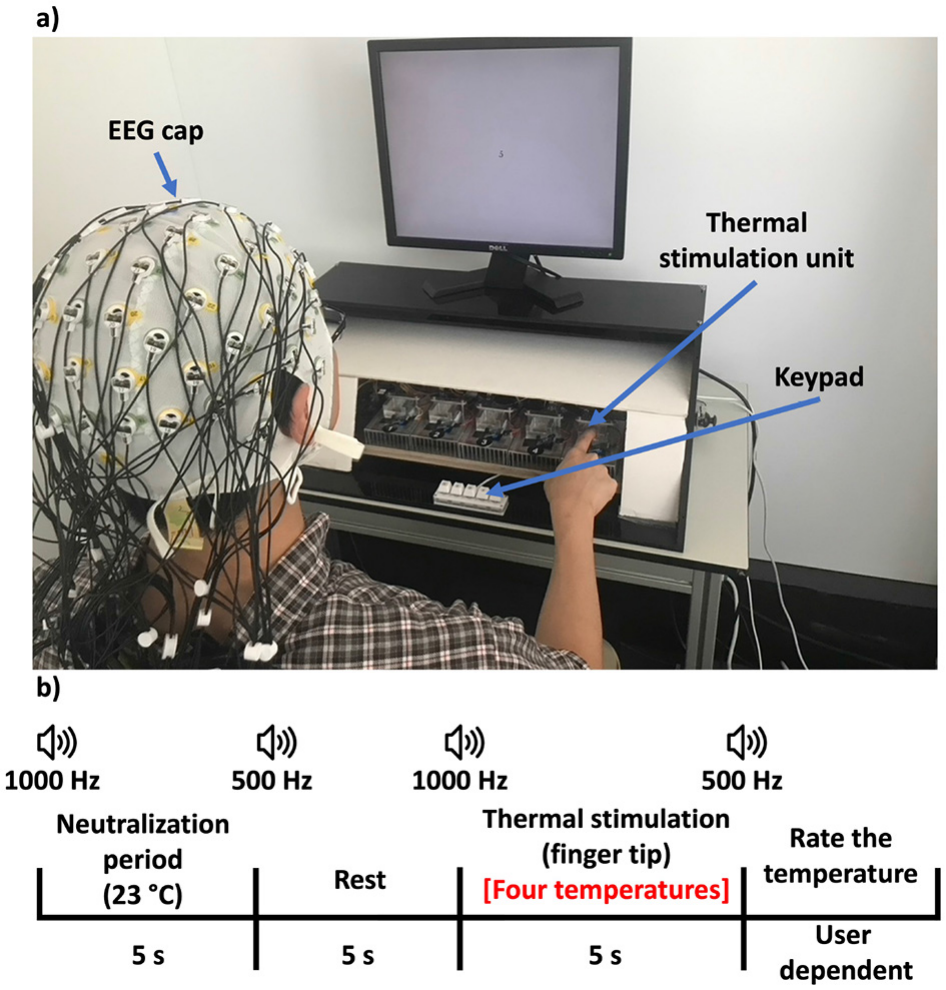
**(a)** Experimental setup of the FTF experience. **(b)** Task and protocol of the FTF experience.

The experimental setup is shown in Figure 4a and the timeline for a single trial is shown in Figure 4b. We describe the task below:

- Neutralization period: Each trial started with a 5 s contact at ~23 °C (ambient temperature), reducing bias from prior stimuli.
- Thermal stimulation: The Peltier pad switched to one of the four target temperatures. Participants actively touched it for ~5 s during which the EEG data were being recorded. The selected target temperatures were 9 °C, 15 °C, 32 °C, or 42 °C, chosen based on psychophysical testing that spanned the relevant cold-to-warm perceptual range reported in prior work (Wanjoo Park and Eid, 2023; Luo et al., 2020).
- Self-report: Afterwards, participants rated perceived temperature (very cold, cold, neutral, hot, and very hot).
- Extended rest: ~30 s of rest allowed the pad to transition to the next temperature and prevented thermal receptor fatigue.
- The process was repeated for a predetermined number of trials (20 trial for each of the four conditions), ensuring balanced coverage of the four thermal stimulation levels.

EEG data were epoched around fingertip contact onset. For additional details on task design and neural analysis, we refer readers to Wanjoo Park and Eid (2023).

#### 2.4.3 Dataset

PS labels consist of four categories (*very cold, cold, hot*, and *very hot*), while the SR labels have five categories (*very cold, cold, neutral, hot*, and *very hot*). To align the categories, all epochs labeled as *neutral* in the SR system were excluded. The remaining epochs have consistent labels across both PS and SR systems, resulting in a four-class classification for this experience.

Table 1 summarizes the details of the four datasets, including participant counts, number of experienced conditions, and the total number of trials recorded.

**Table 1:** Summary of datasets used in the study for each haptic modality.

**Dataset**	**Subjects**	**Conditions**	**# Classes**	**Trials**	**Channels**
DFF	34	4	2	40 × 4 × 34 = 5, 440	60
FVF	29	3	3	100 × 3 × 29 = 8, 700	29
UVF	31	3	3	36 × 3 × 31 = 3, 348	60
FTF	28	4	4	20 × 4 × 28 = 2, 240	60

### 2.5 Labels analysis

**Figure 5:**
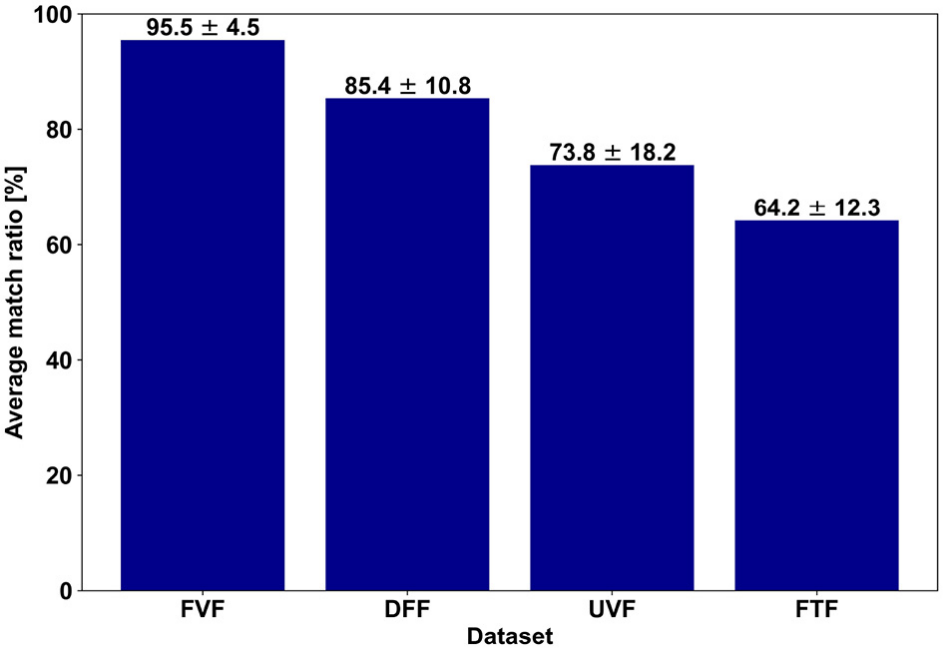
Average match ratios between the PS and SR labels for the four datasets across subjects.

A central question in this study is how often participants' *SR* labels coincided with the *PS* labels. To quantify this, we introduce what we call the *match ratio*, which measures the fraction of trials in which the SR labels exactly agree with the PS label for each subject. Figure 5 shows a bar plot highlighting the average match ratio of all participants for each of the four haptic experiences.

**Figure 6:**
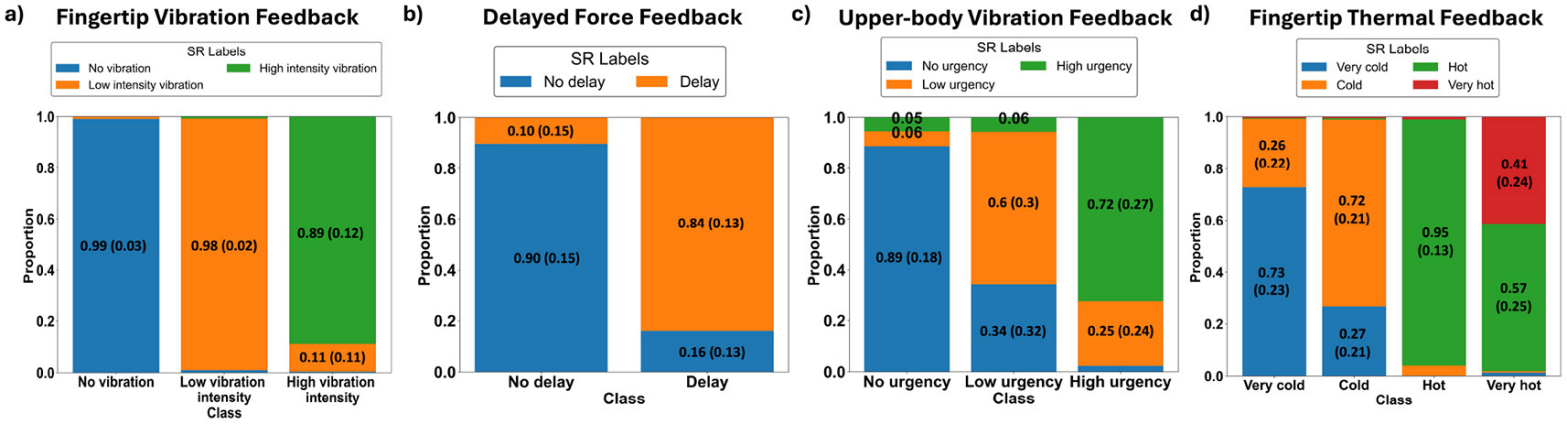
Match ratio across the different classes for each haptic experience averaged across subjects (std. deviation between the brackets). **(a)** Fingertip vibration feedback (FVF). **(b)** Delayed force feedback (DFF). **(c)** Upper-body vibration feedback (UVF). **(d)** Fingertip thermal feedback (FTF).

It can be observed that FVF dataset has the highest match ratio, averaging around 95%. The discrepancy between the PS and SR labels is mostly in the low intensity vibration class. The second highest match ratio is for the DFF dataset (around 85%), where haptic delay is examined. The threshold of haptic delay detection for force-feedback is estimated to be around 85 ms (Haneen Alsuradi and Eid, 2022), and thus, most of the mismatched trials are the ones originally PS-labeled as 120 ms delay (see Figure 6b) (Alsuradi and Eid, 2023; Haneen Alsuradi and Eid, 2022). Next is the UVF dataset, examining perceived urgency, with an average match ratio of around 73%. The FTF dataset has the lowest match ratio, averaging around 64%. Adaptation to a prior temperature or an individual's tolerance can cause confusion, particularly between adjacent levels (e.g., 15 °C mistaken for “very cold” instead of “cold”). Furthermore, the thermal experience is one of the most subjective and personal haptic experiences (Wanjoo Park and Eid, 2023), and thus the observed low match ratio. Figure 6 shows the match ratio between the PS labels and the mapped SR labels per class, for each of the four haptic experiences.

Generally, several factors may contribute to mismatches between PS and SR labels such as the detection threshold for haptic delays or personal thermal experience (Haneen Alsuradi and Eid, 2022; Wanjoo Park and Eid, 2023). Furthermore, factors such as attention, prior experience, and cognitive load further influence how a stimulus is perceived. Additionally, fatigue and expectations can lead to over- or underestimation of stimuli. Ambiguities in linguistic categories, such as “urgent” vs. “very urgent” may also cause inconsistencies in labeling.

### 2.6 Deep learning models

Developing EEG-based classifiers for haptic experience evaluation requires deep-learning architectures that capture both the complex temporal dynamics and the multi-channel spatial patterns inherent in brain signals. In this work, we adopted three state-of-the-art bench-marked models: *ATCNet* (Altaheri et al., 2022), *EEG Inception* (Zhang et al., 2021), and *EEG Conformer* (Song et al., 2022). For each dataset, we trained models separately using PS labels and SR labels. This parallel labeling approach allowed us to systematically assess how the choice of labeling scheme affects classification performance across four distinct haptic experiences.

The three models are designed to address the same challenge of capturing the spatio-temporal nature of EEG data, each doing so in its own unique way. Below, we discuss how these models approach this task with their distinct methodologies.

**ATCNet (Attention Temporal Convolutional Network) (Altaheri et al.**, **2022****)** processes the EEG data in three consecutive steps: the first is signal encoding through several convolutional layers where high-level spatial features are extracted from the raw signal. The second step uses multi-head self-attention layers where the most valuable information in the temporal sequence are extracted. Finally, high-level temporal features are extracted through the use of temporal convolutional network (TCN), improving the overall encoding of the network. ATCNet concludes with fully connected layers that transform these learned representations into class probabilities. The rationale behind the design of ATCNet's architecture lies in EEG's tendency for brief but highly informative activations [for example, event-related potentials or transient oscillations (Bastiaansen et al., 2011)] that might otherwise be overlooked. Through the multi-head attention mechanism, such features can be more effectively captured.

**EEG inception** (Zhang et al., 2021) is inspired by the Inception CNN model (Szegedy et al., 2016) used in computer vision applications. The main rationale of this architecture is based on multi-scale parallel convolutions. Instead of relying on a single kernel size, it applies several of them simultaneously to capture features over different time-frequency scales. After these parallel convolutions, feature maps are concatenated, forming a multi-resolution representation of the data. EEG signals often reflect multiple frequency bands (Bastiaansen et al., 2011) (delta, theta, alpha, beta, and gamma) and can show fast transients alongside slower rhythms. By processing multiple kernel sizes in parallel, EEG Inception could potentially capture short-lived events, like a rapid synchronization or desynchronization following haptic onset, as well as more sustained oscillatory patterns. This makes EEG Inception particularly well-suited for tasks involving subtle yet prolonged phenomena, such as mild changes in thermal experience.

**EEG conformer** (Song et al., 2022) is a hybrid model that merges convolutional operations with transformer-based self-attention, originally inspired by speech and language processing (Vaswani, 2017). Convolutional layers first extract local temporal and spatial features, handling short bursts or focal oscillations. Beyond this local scope, self-attention enables the network to track relationships that may span broader time scales, crucial for stimuli like prolonged thermal stimulation or a continuous force-feedback delay. Positional encoding preserves the ordering of samples within the time series, ensuring that “early” and “late” segments of a trial are recognized as distinct. By blending convolution (for local detail) and attention (for global context), EEG Conformer can capture both immediate sensory events and more extended, possibly multi-second processes.

### 2.7 Training and validation

All the three models were trained based on LOSO cross-validation framework. LOSO is often considered robust for generalization across subjects as it tests the model's ability to predict unseen subjects, which is crucial in personalized applications and brain-computer-interface applications, where individual variability is significant (AlSharabi et al., 2023; Baygin et al., 2023). In LOSO, each subject's data gets the chance to be a test set, while the remaining subjects' data forms the training set. This process is repeated until every individual had been tested exactly once. The final reported accuracy within the manuscript is the average across all subjects.

Before training, *z*-score normalization on a per-channel basis is applied, computing mean and standard deviation using all trials from the training subjects combined and applying the same statistics to the test subject's data, ensuring no test-set leakage. To combat overfitting, we augmented the training set by adding copies of the data with small amount of Gaussian noise (0.5% of the raw amplitude), effectively pushing the model to learn more robust features (yielding a few percent increase in model's performance). In cases of class imbalance (e.g., one SR-labeled category less frequent than others), we applied class weights in the loss function to ensure each class contributed proportionally to the gradient updates. This is done by computing weights dynamically for each LOSO cross-validation fold such that the weight assigned to each condition is inversely proportional to its frequency in the training data. Although our SR-labeled datasets exhibit only mild imbalance (Krawczyk, 2016) (the imbalance ratio, which is the largest class divided by smallest, is always below 4), we applied this procedure to avoid any performance discrepancies attributable to class distribution rather than label quality.

All models were trained for 50 epochs using a batch size of 32. We started with a learning rate of 1 × 10^−3^ and employed a *plateau* scheduler; if the validation-set loss showed no improvement after a patience window of 10 epochs, the learning rate was reduced by half. We used the AdamW optimizer, which combines Adam's adaptive momentum with weight-decay regularization to avoid overfitting. During backpropagation, we clipped gradients (max-norm of 1.0) to prevent potential instability due to large updates.

Accuracy was computed as the percentage of correctly classified trials. Per-class accuracy values were also calculated for the purpose of clarifying which classes were most impacted by the label choice. Because the underlying EEG data and hyperparameters were identical, the observed difference in accuracy should be attributed almost entirely to the labeling choice.

## 3 Results

### 3.1 Overall classification results

Table 2 summarizes the classification outcomes for the four haptic experiences under both labeling schemes for the three considered deep learning models. Across all haptic experiences, and for the three considered architectures (ATCNet, EEG Conformer, and EEG Inception), models trained with PS labels clearly demonstrate better or at least equivalent performance compared to their SR counterparts. Furthermore, the ATCNet model appears to perform the best in most haptic experiences and is, therefore, selected for further analysis. A statistical comparison was performed to evaluate each of the model's classification accuracy under both schemes. As shown in Table 2, ATCNet models trained with PS labels performed significantly better for DFF, FVF, and UVF, while no significant difference was observed for the FTF dataset.

**Table 2:** Models classification performance under PS vs. SR labeling schemes.

**Metric**	**Dataset**	**ATCNet**		**EEG conformer**		**EEG inception**	
		**PS**	**SR**	**PS**	**SR**	**PS**	**SR**
Accuracy	DFF	74.6 ± 8.6^***^	62.8 ± 9.0	69.7 ± 8.1^***^	58.3 ± 7.4	73.3 ± 8.3^***^	63.5 ± 10.9
	FVF	55.7 ± 5.6^***^	52.4 ± 5.5	46.7 ± 5.2	46.0 ± 4.5	52.1 ± 6.0^***^	49.2 ± 5.7
	UVF	82.9 ± 8.9^***^	60.8 ± 15.4	75.9 ± 7.8^***^	53.5 ± 11.4	80.2 ± 8.2^***^	56.8 ± 12.4
	FTF	40.7 ± 8.7	37.4 ± 7.2	43.1 ± 7.0^***^	35.0 ± 6.8	43.1 ± 11.0	40.0 ± 9.9
F1 score	DFF	69.0 ± 9.5^***^	59.8 ± 8.8	63.3 ± 8.1^***^	55.3 ± 5.9	67.5 ± 8.9^***^	57.8 ± 9.7
	FVF	55.0 ± 5.8^***^	51.4 ± 5.6	45.7 ± 5.3	44.6 ± 4.7	51.3 ± 6.2^***^	48.1 ± 6.1
	UVF	82.7 ± 9.3^***^	57.5 ± 16.8	75.7 ± 8.0^***^	50.4 ± 12.3	80.0 ± 8.5^***^	52.4 ± 14.8
	FTF	33.8 ± 7.0^*^	30.6 ± 7.0	35.9^**^± 7.7	30.3 ± 7.0	35.9 ± 9.7^**^	30.0 ± 6.8

Reported values are mean ± standard deviation across subjects. Statistically significant differences between PS-trained and SR-trained models are indicated (^*^p < 0.05; ^***^p < 0.001; ^**^p < 0.01; paired *t-*test, Bonferroni correction for multiple comparisons across models).

**Figure 7:**
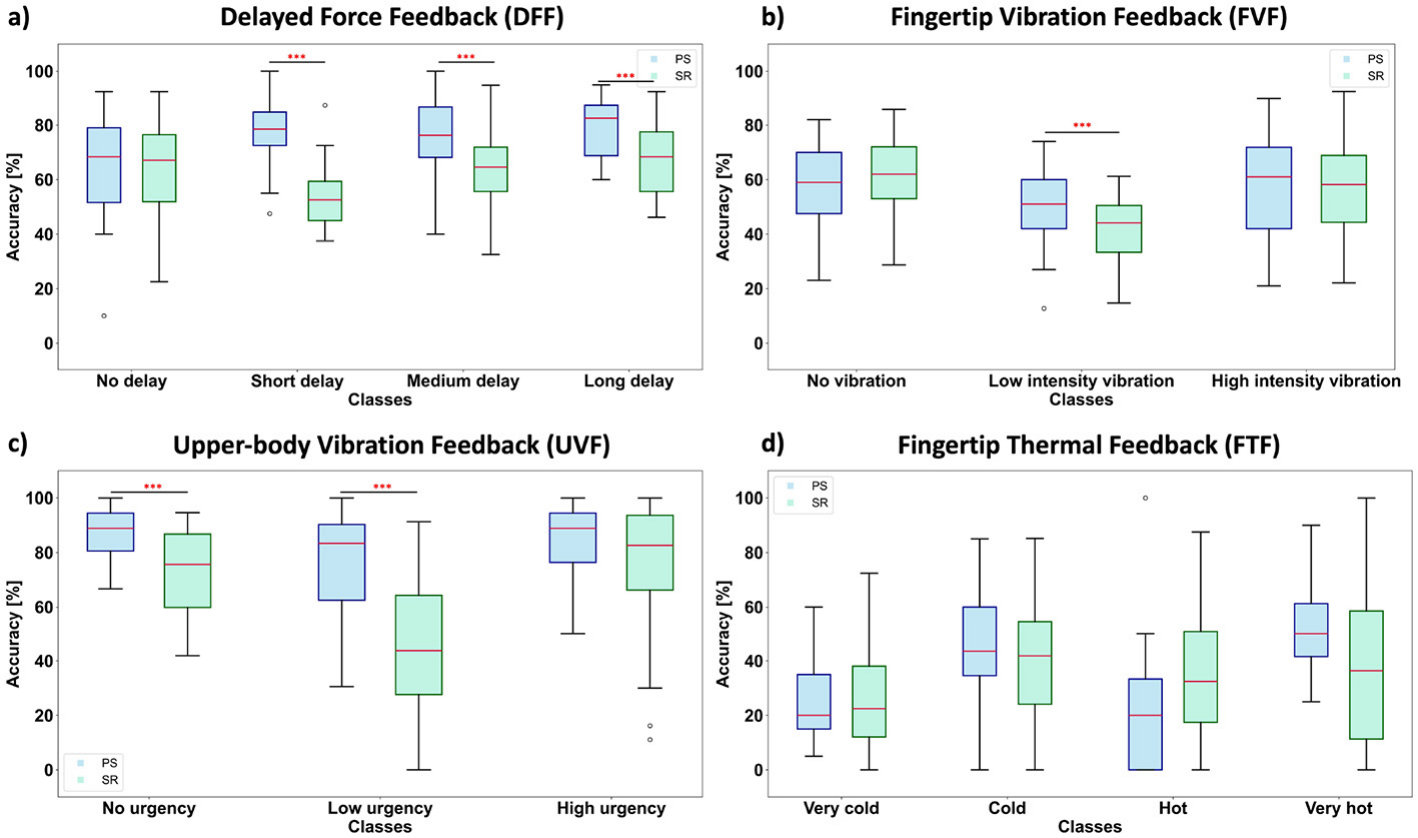
Boxplots comparing per-class accuracy values for the ATCNet model under PS and SR labeling schemes, across the four haptic experiences. Paired *t*-test, Bonferroni corrected: ^***^p < 0.001, ^**^p < 0.01, ^*^p < 0.05. **(a)** Delayed force feedback (DFF). **(b)** Fingertip vibration feedback (FVF). **(c)** Upper-body vibration feedback (UVF). **(d)** Fingertip thermal feedback (FTF).

In the DFF experience, the PS-trained models outperformed the SR-trained ones by a substantial margin (see ATCNet at 74.6% vs. 62.8%). This observation aligns with psychophysical evidence suggesting that borderline delays (~120–250 ms) are more difficult to identify (Haneen Alsuradi and Eid, 2022) and, therefore, inconsistently labeled by participants, leading to reduced performance for SR-trained models (see Figure 7a, short delay).

A similar pattern is observed in the UVF dataset, where the PS-trained model shows a 22.1% increase in the average accuracy compared to the SR-trained model. Previous vibrotactile experiments on the torso (Schmidt and Carter, 2023) revealed that participants frequently misjudged moderate differences in amplitude or vibrotactile motor count, reflecting variability in sensitivity across subjects, which could have caused lower performance for SR-trained models (see Figure 7c, low urgency).

As for the FVF experience, the match ratio between the PS and SR labels was around 95% (see Figure 5), which explains the relatively smaller difference between the PS-trained and SR-trained models' performance.

**Figure 8:**
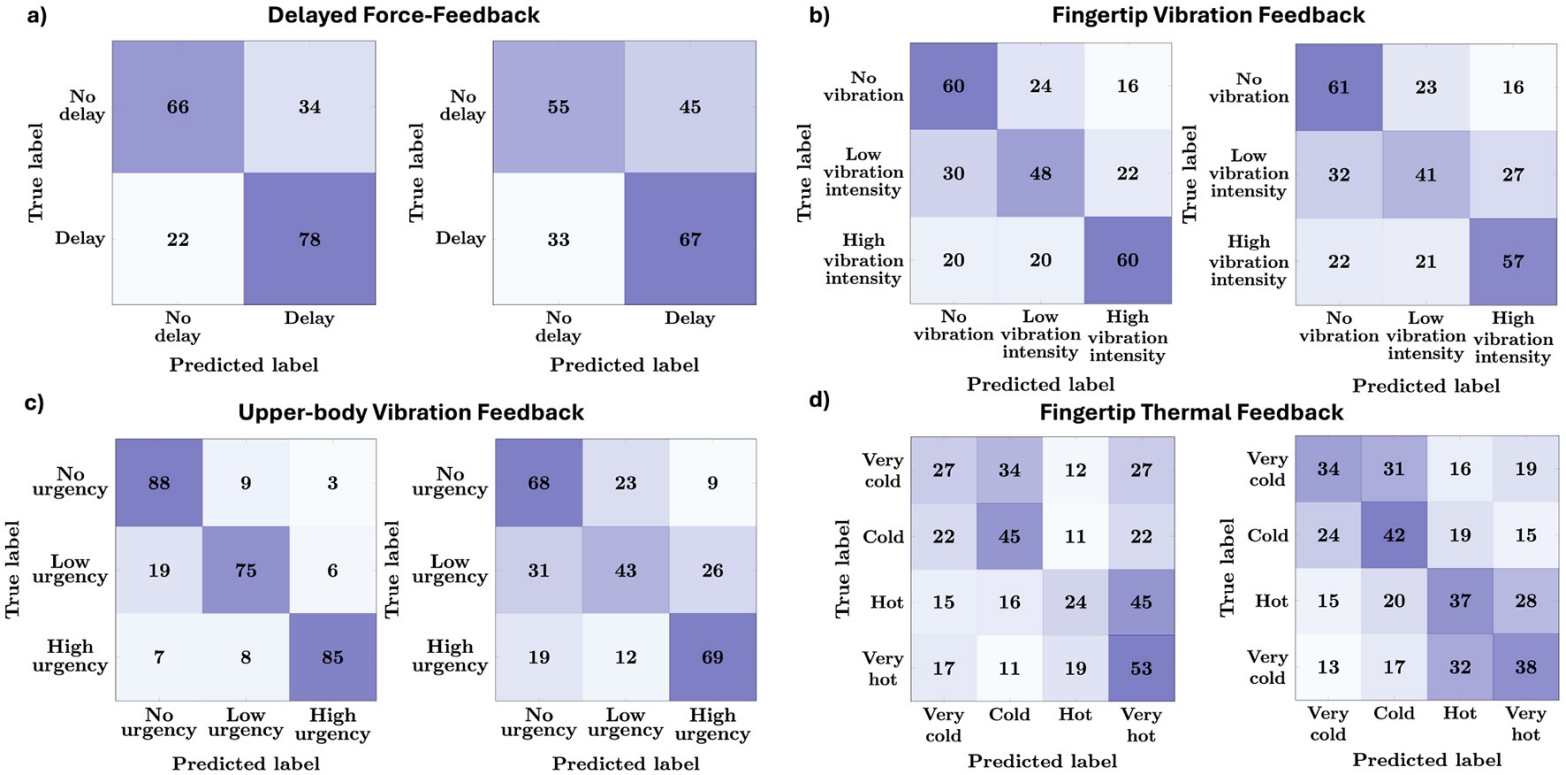
ATCNet model confusion matrices for the four haptic modalities (left: PS-trained model, right: SR-trained model). **(a)** Delayed force feedback. **(b)** Fingertip vibration feedback. **(c)** Upper-body vibration feedback. **(d)** Fingertip thermal feedback.

Thermal classification, consistent with previous thermal psychophysics, proved particularly challenging (Karmakar et al., 2023; Jones and Berris, 2002). For PS-trained models, accuracy values remained in the 40%–43% range for a four-class classification. This relatively poor performance in FTF is possibly due to the high similarity between the very cold/cold classes and the very hot/hot classes, as evident from the confusion matrix presented in Figure 8d. Another source of confusion may stem from the perceptual overlap between extreme temperatures (very cold/very hot, often close to pain thresholds) and their milder counterparts (cold/hot), which are less intense and non-extreme. When data is labeled subjectively, performance dipped a few points further. The precise boundaries between, *cold* vs. *very cold* or *hot* vs. *very hot* are subject to wide variation and adaptation, which aggravates label subjective differences (Jones and Berris, 2002). Those classes were confused by the model, for both, PS-trained and SR-trained versions, indicating subtle neural differences.

### 3.2 Per-class accuracy analysis

Figure 7 depicts the per-class accuracy values for the ATCNet model trained under the PS and SR labeling schemes. Examining the model's performance on each of the haptic experiences reveals a variation in the reliability of the model in evaluating the different levels of a particular experience. The figure also shows how these variations change when subjective labels are replaced by physically defined labels. This is also evident from the set of confusion matrices shown in Figure 8 for the four haptic experiences under both labeling schemes.

For the DFF experience, it is clear that the *no delay* class is well classified for both labeling schemes. On the other hand, intermediate delays, labeled as *short delay* or *medium delay*, are better classified under the PS scheme (Figure 7a). Although participants were exposed to four distinct delay levels (0, 120, 250, and 400 ms), their subjective reports were collected in a binary manner (delay/no delay). Accordingly, Figure 7a evaluates binary classification performance separately for each physical delay condition, revealing that the near-threshold delays (120 ms and 250 ms) are the most challenging for SR-trained models.

For the FTF experience, which involves slow-response temperature cues, both PS and SR labeling produced comparatively low accuracy values overall. However, the PS-trained ATCNet showcased more stability and better performance (higher mean, narrower interquartile) for the extreme conditions (*very cold, very hot*). Intermediate temperatures seem to be confused for both labeling schemes (Figures 7d, 8d).

For the FVF experience, the boxplots indicate that PS-trained models outperform SR-trained models, particularly for low vibration intensity, which represents an intermediate condition. In contrast, for far-from-threshold vibration intensities (*no vibration* or *high vibration intensity*), the label match ratio is relatively high, resulting in comparable performance between the PS-trained and SR-trained models (Figures 7b, 8b).

The UVF experience exhibited the most pronounced gap in performance between PS-trained and SR-trained models. The PS-trained model have boxplots that demonstrate higher median accuracy, coupled with a tighter interquartile range for all three classes. However, the most pronounced difference lies in the intermediate class (low urgency) as shown in Figures 7c, 8c.

### 3.3 Match ratio and classification accuracy

**Figure 9:**
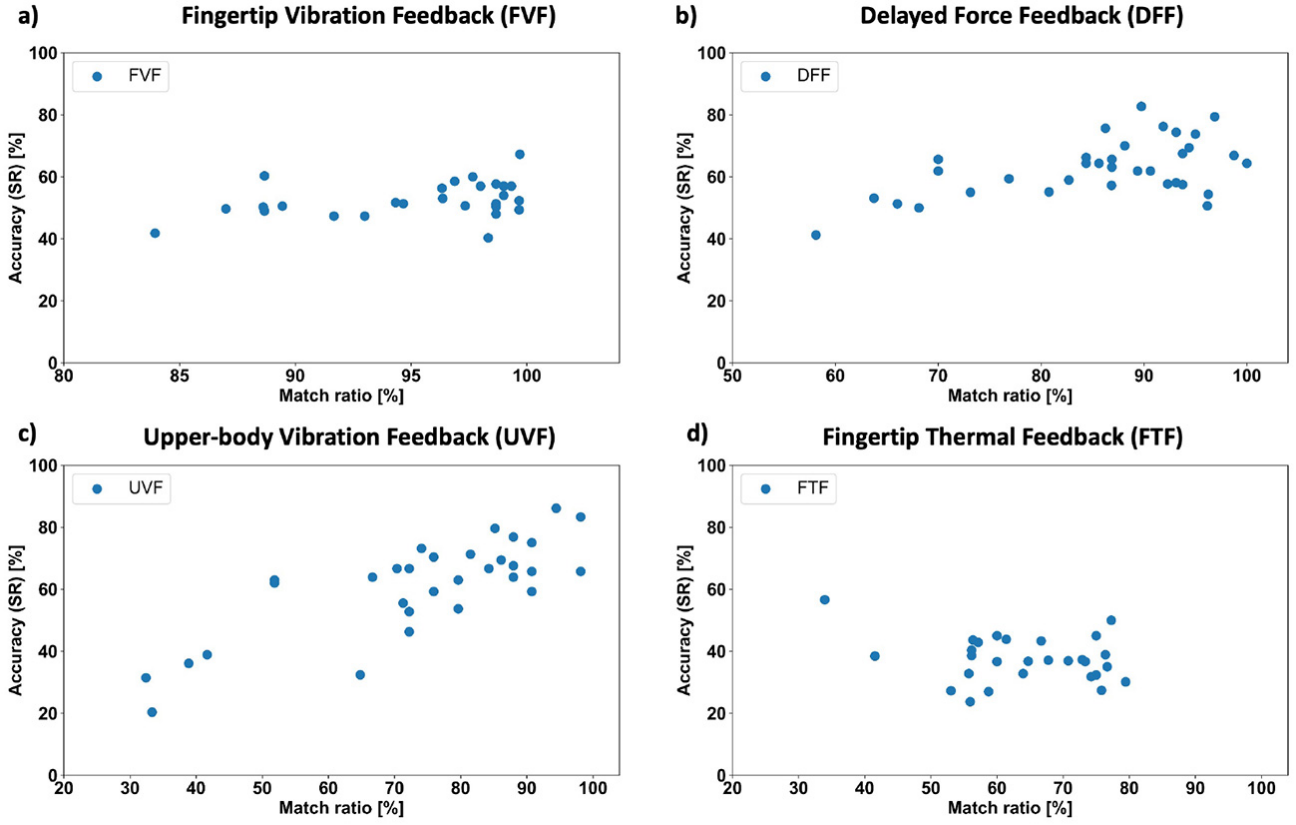
Scatter plot highlighting the correlation between match ratio index and SR-trained ATCNet model for the **(a)** Fingertip vibration feedback (FVF), **(b)** Delayed force feedback (DFF), **(c)** Upper-body vibration feedback (UVF), and **(d)** Fingertip thermal feedback (FTF). Each data point represents a single test subject.

The discrepancy between SR and PS labels is numerically captured by the introduced match ratio index. Figure 9 presents correlation plots between the match ratio index and the SR-based classification accuracy for each of the four haptic experiences. A positive but non-significant correlation was observed for FVF experience between the match ratio index and the SR-trained ATCNet accuracy (*t* = 1.84, *p* = 0.076; Pearson's *r*(27) = 0.334, 95% CI [−0.037, 0.624]), indicating only a weak association. In contrast, DFF showed a significant moderate correlation (*t* = 3.64, *p* = 9.56 × 10^−4^; Pearson's *r*(32) = 0.541, 95% CI [0.248, 0.743]), suggesting that higher match ratios reliably correspond to improved SR-based model performance. UVF exhibited the strongest effect, with a robust and statistically significant correlation (*t* = 6.98, *p* = 1.13 × 10^−7^; Pearson's *r*(29) = 0.792, 95% CI [0.608, 0.895]), indicating a clear relationship between match ratio and SR-trained accuracy. Finally, no significant correlation was found for FTF (*t* = −1.30, *p* = 0.207; Pearson's *r*(26) = −0.246, 95% CI [−0.567, 0.140]), suggesting no meaningful linear association for this experience.

## 4 Discussion

The results in Sections 3.1, 3.2 illustrate how using PS versus SR labels relates EEG-based classifiers differently to the physical stimulation and to participants' reported experiences. In all four haptic experiences examined (DFF, FVF, UVF, and FTF), PS-labeling offered systematically higher or at least equivalent performance relative to SR.

The largest performance gaps between PS-trained and SR-trained models emerged in mid-range classes, such as moderate vibration intensities, low urgency levels, or intermediate delays (120–250 ms). Prior psychophysical studies confirm that human perception near detection thresholds can vary substantially across individuals (Mueller and Grunwald, 2023; King et al., 2010). While SR labeling can capture user-specific nuances, it also amplifies inter-subject variation in these delicate ranges, introducing label inconsistencies that can degrade model training when models are trained at the group level, as in our LOSO cross-subject setting (Schmidt and Carter, 2023; Haneen Alsuradi and Eid, 2022; Wanjoo Park and Eid, 2024). By contrast, PS-labeled data link EEG epochs to physically imposed parameters that are consistent across subjects, thereby mitigating variability due to individual differences and uncertainty in the group-level training set and stabilizing the learned features.

Other sources of discrepancy between SR and PS labels (apart from the individual differences at near-threshold conditions) stem from lack of attention, or sensory illusion, both of which are unavoidable types of human errors at the cognition level. These errors can occur even under controlled experimental conditions and may significantly affect SR-labeled data. For instance, participants may momentarily lose focus or misinterpret stimuli due to cognitive fatigue.

While ATCNet demonstrated the highest performance, it remains clear that under our group-level LOSO setting, PS labeling benefited EEG Conformer and EEG Inception in nearly equal measure. This supports the observation that label consistency across subjects may outweigh modest differences in network design for EEG data decoding (Schmidt and Carter, 2023). In practice, stable labels enable each model to learn relevant spatiotemporal features from pooled multi-subject data. For example, ATCNet's attention mechanism, is adept at focusing on critical temporal windows, but if many of the EEG data epochs are inconsistently labeled or uncertain, particularly for SR labels near-threshold conditions, the advantage diminishes. As for the models' comparisons, ATCNet's dilated convolutions and attention likely made it adept at capturing rapid oscillations in vibration (FVF/UVF) and transient responses in delayed force-feedback (DFF). EEG Inception's multi-scale filters likely picked up slow thermal potentials (FTF) and mid-latency activity in moderate conditions. EEG Conformer's hybrid architecture likely learned fine sensory features and long-range dependencies relevant to upper-body vibration (UVF).

Although PS labels showed more stable performance at the group level in our LOSO setting, PS-trained models are best viewed as a first-stage representation that captures the structured, controllable aspects of the stimulus space. By learning systematic relationships between physical parameters and resulting sensory experiences, PS-based models can later be adapted with personalized SR labels for subject-specific use. Alternatively, approaches such as semi-supervised learning (Van Engelen and Hoos, 2020), domain adaptation (Farahani et al., 2021), or real-time label correction (Zheng et al., 2021) present promising strategies for integrating subjective experiences into the training pipeline while still leveraging this PS-based backbone. Furthermore, combining EEG with other physiological signals, such as EMG or GSR, could aid in resolving ambiguity in mid-range (around the threshold) classes, where SR-labels variability is most pronounced.

## 5 Conclusion

This study contributes to the development of cognitive haptic interfaces by investigating how different labeling strategies (PS vs. SR) influence EEG-based classification of haptic experiences using deep learning models. Across four distinct haptic experiences (DFF, FVF, UVF, and FTF) and three deep learning architectures (ATCNet, EEG Inception, and EEG Conformer), PS labels yielded more stable and generally higher classification performance than SR labels in our group-level LOSO setting, with the largest differences at near-perceptual-threshold stimulus conditions.

For future work, several directions could be pursued. From the perspective of labels, exploring multi-label classification approaches may offer valuable insights. Additionally, other haptic experiences (such as haptic jitter, vibrations on different body parts, painful versus non-painful thermal stimulation, or variations in vibration frequency) could be investigated and subjected to similar testing. Finally, moving beyond training independent networks for specific haptic modality, a valuable step would be to develop a global haptic experience identifier capable of determining both the modality and the intensity of a given EEG input.

## Data Availability

The data that support the findings of this study are publicly available in the Open Science Framework at https://osf.io/7up4z/. The analysis scripts and implementation code are available in a public GitHub repository at https://github.com/Yonas650/PSvsSR/.
